# Next-generation immuno-oncology agents: current momentum shifts in cancer immunotherapy

**DOI:** 10.1186/s13045-020-00862-w

**Published:** 2020-04-03

**Authors:** Chongxian Pan, Hongtao Liu, Elizabeth Robins, Wenru Song, Delong Liu, Zihai Li, Lei Zheng

**Affiliations:** 1 Chinese American Hematologist and Oncologist Network, New York, NY USA; 2 University of California, Davis, CA USA; 3 University of Chicago, Chicago, IL USA; 4 Pelotonia Institute for Immuno-Oncology, The Ohio State University, Columbus, OH USA; 5 Kira Pharmaceuticals, Cambridge, MA USA; 6 New York Medical College, Valhalla, NY USA; 7 Johns Hopkins University, Baltimore, MD USA

**Keywords:** Neoantigen, Immune checkpoint inhibitor, Tumor microenvironment, PD-1, PD-L1, CTLA-4, CAR-T

## Abstract

Cancer immunotherapy has reached a critical point, now that immune checkpoint inhibitors and two CAR-T products have received market approval in treating 16 types of cancers and 1 tissue-agnostic cancer indication. Accompanying these advances, the 2018 Nobel Prize was awarded for the discovery of immune checkpoint pathways, which has led to the revolution of anti-cancer treatments. However, expanding the indications of immuno-oncology agents and overcoming treatment resistance face mounting challenges. Although combination immunotherapy is an obvious strategy to pursue, the fact that there have been more failures than successes in this effort has served as a wake-up call, placing emphasis on the importance of building a solid scientific foundation for the development of next-generation immuno-oncology (IO) agents. The 2019 China Cancer Immunotherapy Workshop was held to discuss the current challenges and opportunities in IO. At this conference, emerging concepts and strategies for IO development were proposed, focusing squarely on correcting the immunological defects in the tumor microenvironment. New targets such as Siglec-15 and new directions including neoantigens, cancer vaccines, oncolytic viruses, and cytokines were reviewed. Emerging immunotherapies were discussed in the areas of overcoming primary and secondary resistance to existing immune checkpoint inhibitors, activating effector cells, and targeting immunosuppressive mechanisms in the tumor microenvironment. In this article, we highlight old and new waves of IO therapy development, and provide perspectives on the latest momentum shifts in cancer immunotherapy.

## Introduction

Cancer immunotherapy has been a game changer in cancer treatment since the approval of the immune checkpoint inhibitor (ICI) ipilimumab in 2011. Currently, 11 immune checkpoint inhibitors (Table 1) and 2 chimeric antigen receptor T cell (CAR-T) products have been approved in treating 16 types of malignant diseases and 1 tissue-agnostic indication. In 2018, one half of the Nobel Prize in Physiology or Medicine was awarded to James Allison, who conceptualized cancer immunotherapy by targeting the immunosuppressive signal mediated by Cytotoxic T Lymphocyte-Associated Protein 4 (CTLA-4) [1, 2]. This conceptual breakthrough led to the subsequent revolutionary development of immune checkpoint inhibitors (ICIs). In addition, co-Nobel Prize awardee Tasuko Honjo showed that a basic mechanism of activation-induced cell death in lymphocytes is mediated by Programmed Cell Death 1 (PD-1) [3]. Honjo subsequently demonstrated that the PD-1 pathway is an important negative regulator of T cell function [4–6].

**Table 1:** Immune checkpoint inhibitors and their US FDA/EMA/China NMPA approved indications

Immune checkpoint inhibitor	Targets	US FDA/EMA approved indications	China NMPA approved indications
Pembrolizumab	PD-1	Melanoma, non-small cell lung cancer, head and neck cancer, Hodgkin’s lymphoma, urothelial carcinoma, MSI-H/dMMR* colorectal cancer, MSI-H/dMMR cancers, gastric cancer, cervical cancer, hepatocellular carcinoma, Merkel cell carcinoma, renal cell carcinoma, small cell lung cancer, esophageal carcinoma, endometrial cancer	Melanoma, non-small cell lung cancer
Nivolumab	PD-1	Melanoma, non-small cell lung cancer, renal cell carcinoma, Hodgkin’s lymphoma, head and neck cancer, urothelial carcinoma, MSI-H/dMMR colorectal cancer, hepatocellular carcinoma, small cell lung cancer	Non-small cell lung cancer
Atezolizumab	PD-L1	Urothelial cancer, non-small cell lung cancer, breast cancer, small cell lung cancer	Non-small cell lung cancer
Durvalumab	PD-L1	Urothelial carcinoma, non-small cell lung cancer	
Avelumab	PD-L1	Merkel cell carcinoma, urothelial carcinoma, renal cell carcinoma	
Cemiplimab	PD-1	Cutaneous squamous cell carcinoma	
Ipilimumab	CTLA4	Melanoma, metastatic, renal cell carcinoma, MSI-H/dMMR colorectal cancer	
Toripalimab	PD-1		Melanoma
Sintilimab	PD-1		Hodgkin’s lymphoma
Camrelizumab	PD-1		Hodgkin’s lymphoma
Tislelizumab	PD-1		Hodgkin’s lymphoma

*Microsatellite instability high (MSI-H) or mismatch repair deficient (dMMR)

While the discoveries made by Allison and Honjo are truly seminal, the IO revolution, like any other scientific breakthroughs, has taken a “village”. Lieping Chen, for example, first cloned Programmed Cell Death 1 Ligand 1 (PD-L1, also known as B7-H1) [7], showed its inhibitory function [8, 9], and indicated that blocking this pathway may have therapeutic potential [9]. Other notable scientific contributors include Gordon Freeman, who collaborated with Honjo to establish the receptor-ligand relationship between PD-1 and PD-L1 [5]; Pierre Goldstein, who first cloned CTLA-4 [10]; and Jeffrey Bluestone [11, 12], Tak Mak [13], and Arlene Sharpe [12], who demonstrated the inhibitory function of CTLA-4. The IO village has also included many clinical investigators, who masterfully designed and completed ICI clinical trials and taken ICIs into today’s standard clinical practice [14]. Having witnessed the flourishing of cancer immunotherapy, the Chinese American Hematologist and Oncologist Network (CAHON), in partnership with the China National Medical Product Administration (NMPA), and later joined by Tsinghua University, have organized the annual China Cancer Immunotherapy Workshop since 2017 to provide update and education to physicians, scientists, and drug developers [15]. The fifth China Cancer Immunotherapy Workshop was held in Tianjin on June 29‑30, 2019, and it proved again to be an international forum on the discussion of the cutting edge of cancer immunotherapy. There were 4 major themes in the 2019 conference. The first theme was centered on current challenges in ICI development, and new visions for the future of this field. The second one focused on the development and application of cell therapy, where new IO agents continue to rapidly emerge. The third theme featured new immunotherapy strategies that are driven by advancements in basic immunology research. The last theme highlighted regulatory challenges and solutions in clinical research and development of cancer immunotherapeutics by experts from the China NMPA, the United States Food and Drug Administration (US FDA), and the European Medicines Agency (EMA).

Herein, we use the program of the 2019 China Cancer Immunotherapy Workshop as the general framework to critically review the most recent conceptual shifts and therapeutic advancements in the increasingly exciting and complex IO field.

## A current perspective on the anti-cancer immune response

Challenges in improving the efficacy of existing immunotherapies, and the development of new ones, have led to a deeper appreciation of understanding the mechanisms underlying an effective anti-cancer immune response, as well as the “defects” that are responsible for the lack of an effective anti-cancer immune response in cancer patients.

### The cancer-immunity cycle

**Fig. 1:**
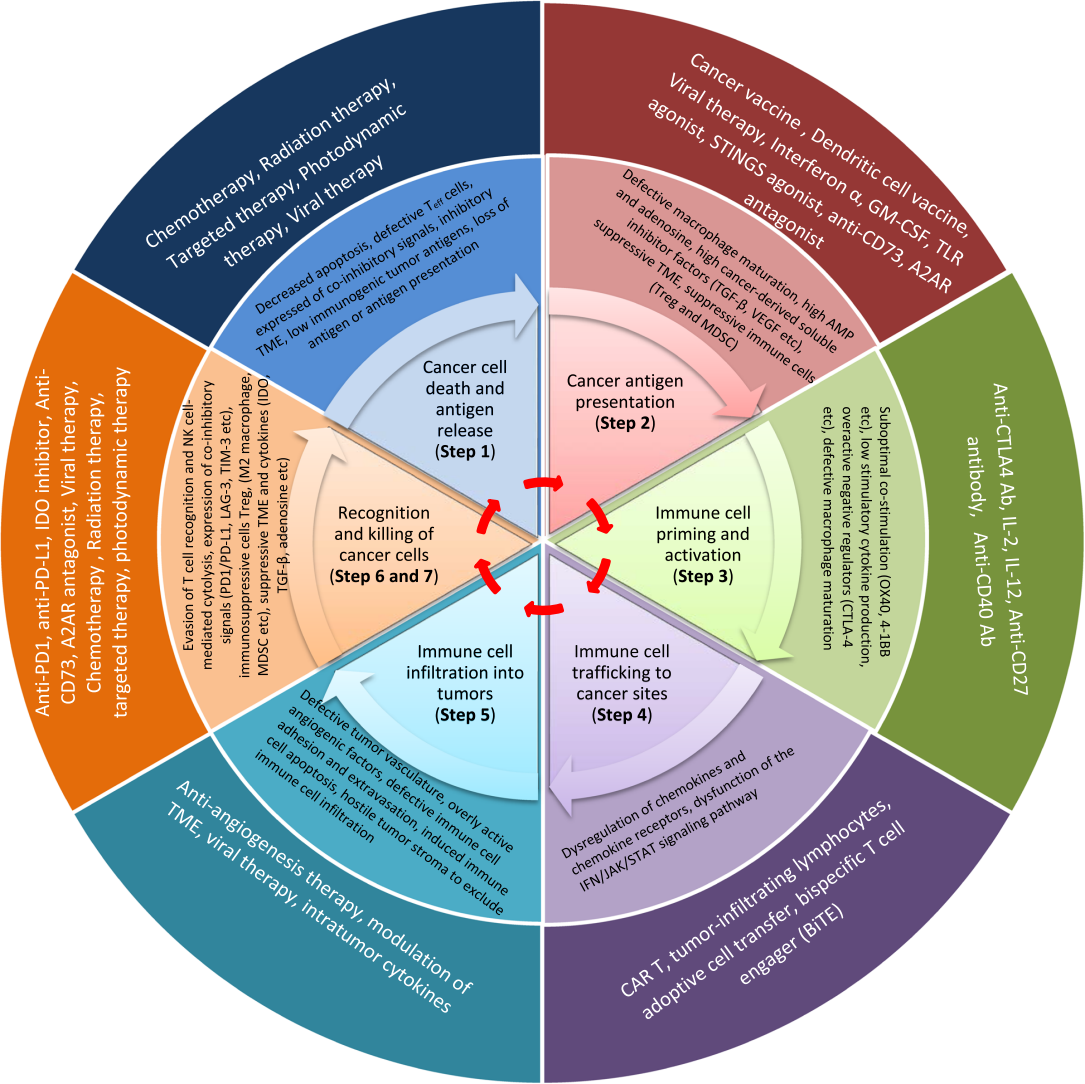
The cancer-immunity cycle, immune-resistant mechanisms and strategies for anti-cancer immunotherapy. The anti-cancer immunity cycle (innermost circle) starts when cancer cells release tumor antigens. Antigen-presenting cells take up tumor antigens and present antigen-derived peptides to immune cells, which in turn activate the immune cells to migrate through the circulation, enter tumor sites, and kill cancer cells. The death of cancer cells induces the release of additional tumor antigens, which initiates another cancer-immunity cycle. The immune system has developed complex negative feedback loops to rein in the anti-pathogen response. These negative feedback loops have been exploited by cancer cells to evade anti-cancer immunity (middle circle). Current anti-cancer immunotherapy approaches (outermost circle) have been targeting and harnessing various mechanisms along this cancer-immunity circle. There are two major approaches for cancer immunotherapy: (1) the enhancement approach, which aims to augment “normal” anti-cancer immune mechanisms. Strategies in this category range from the traditional non-specific enhancement of IL-2 signaling to the more recent cancer-specific CAR-T cell therapy; and (2) the normalization approach, which aims to restore defective anti-cancer immunity in the tumor microenvironment. Strategies include FDA-approved immune checkpoint inhibitors and other drugs in development (e.g., inhibitors of the adenosine pathway)

We present a model of the anti-cancer immunity “cycle” (Fig. 1, innermost circle**)** [16], which provides a summary of our scientific knowledge on each step of an effective anti-cancer immune response. The cycle starts when tumor antigens are recognized by the immune system. Genomic instability/mutation is 1 of the 2 enabling characteristics of cancer [17]. All cancers, regardless of their tissue origin(s), harbor genetic alterations that range from a few mutations in pediatric malignancies to dozens or hundreds in adult cancers [18]. These non-synonymous DNA alterations can give rise to proteins that differ from the proteins expressed in normal cells, i.e., tumor antigens. As a second enabling characteristic, some cancers express non-mutation-associated tumor antigens, such as proteins normally expressed in immune-privileged sites, viral proteins, or proteins encoded by endogenous retroviral genes. When these antigens are taken up and processed by professional antigen-presenting cells (APCs), the APCs migrate to secondary lymphoid organs and activate naïve T cells in concert with a highly-coordinated hierarchy of co-stimulatory signals, such as the CD28/B7-1/2-mediated signal. To achieve homeostasis and prevent over-reaction to non-self antigens, the immune system has also developed highly coordinated negative feedback circuits. CTLA-4 is one of the major negative regulators of the T cell-mediated immune response. CTLA-4 expression is rapidly upregulated upon T cell receptor (TCR) engagement [19], allowing it to outcompete CD28 for ligation by B7-1/2, and thereby negatively regulate T cell activation and effector function.

Once activated, effector T cells traffic into the body systemically, infiltrate the cancer site(s), recognize cancer cells expressing tumor antigen-derived peptides presented by Major Histocompatibility Complex (MHC), and kill target cancer cells. In turn, cancer cells release neoantigen(s) which are cross-presented by APCs, leading to further amplification of the anti-cancer immune response by allowing priming and activation of more T cells to recognize and attack the tumor [20]. Just as in any immune response, the final stage of the anti-cancer response is regulated by a complex network of stimulatory and inhibitory accessory pathways. The PD-1/PD-L1 pathway is one of the major inhibitory pathways. Engagement of the TCR with its cognate antigen-MHC complex, together with cytokine stimulation (e.g., IL-2 stimulation), induces the expression of PD-1. Engagement of PD-1 with PD-L1 on target cells inhibits T cell proliferation and IL-2 production, dampening the immune response. Thus, a rational combination immunotherapy must be aimed at coordinated facilitation of T cell activation and effector function, along with coordinated suppression of inhibitory T cell mechanisms.

### The immune microenvironment of the tumor

**Fig. 2:**
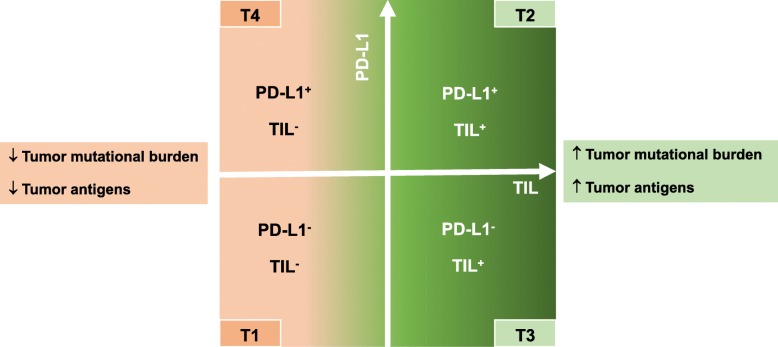
TIME classification based on PD-L1 expression and infiltration of immune cells, mainly tumor-infiltrating lymphocytes (TIL), in the tumor microenvironment. TIME, tumor immunity in the microEnvironment

Study of a Tumor Immunity in the MicroEnvironment (TIME) classification system can be used as the first step in assessing anti-cancer immunity and determining underlying tumor resistance mechanisms. TIME classification is based on two major factors: (1) tumor expression of PD-L1, and (2) the presence of immune cell infiltration, mainly tumor-infiltrating lymphocytes (TIL) (Fig. 2) [21–23]. Correspondingly, 4 distinct TIME subtypes can be described [21]: T1 (PD-L1^−^, TIL^−^), T2 (PD-L1^+^, TIL^+^), T3 (PD-L1^−^, TIL^+^), and T4 (PD-L1^+^, TIL^−^).

In cancers with no immune cell infiltration (T1 or T4 TIME), no anti-cancer immunity exists at the cancer site(s), suggesting defects in cancer antigen release (Cancer-Immunity Cycle Step 1), presentation (Cancer-Immunity Cycle Step 2), immune cell priming and activation (Cancer-Immunity Cycle Step 3), or trafficking of immune cells into cancer sites (Cancer-Immunity Cycle Step 4). In these cases, normalization of cancer immunity using anti-PD1/PD-L1 therapy may not work, since no cancer immunity exists to be de-repressed. On the other hand, the majority of solid tumors (approximately 70%) have a T4 TIME, which underscores the importance of developing rational IO combinations to address both a lack of effector cell infiltration and the presence of non-PD-L1/PD-1 immunosuppressive components. Furthermore, T1 or T4 TIME tumors often exhibit low levels of tumor mutation burden and tumor antigens. For example, androgen-dependent prostate cancer usually presents with a T1 or T4 TIME, with little lymphocyte infiltration. In other cases, physical barriers can inhibit TIL infiltration such as in pancreatic cancer, even though an anti-cancer immune response emerges in some tumors, an immune-excluded phenotype is commonly observed because the desmoplastic stroma precludes the immune cells from penetrating into the tumor.

In cancers with immune cell infiltration into the TIME (T2 and T3), an anti-cancer immune response exists. However, the immunosuppressive TIME inhibits the activity of effector immune cells from killing cancer cells. Absence of PD-L1 in T3 (PD-L1^−^, TIL^+^) suggests that the suppression of anti-cancer immunity is largely mediated by mechanisms other than the PD1/PD-L1 pathway. Interestingly, the T3 TME only exists in approximately 10% of solid tumors. Thus, agents that target alternative co-inhibitory pathways, such as anti-CTLA-4 antibody and anti-LAG3 antibody, may be effective in cancers with a T3 TIME. On the other hand, while TIL are present in T2 and T3 TIMEs, their location and functional capacity may be crucial [24–27]. The immune-inflamed phenotype of T lymphocytes is often accompanied by myeloid and monocytic cells and TIL infiltration into the tumor sites, while the immune-excluded phenotype is characterized by immune cell retention in the stroma that surrounds nests of tumor cells, but does not penetrate into the tumor parenchyma. Cancers with high mutation burdens, such as melanoma, lung, and bladder cancer, can induce a strong anti-cancer immune response, and therefore present with T2 or T3 TIME phenotypes with abundant lymphocyte infiltration. In this manner, TIME classification complements the cancer immunity cycle, stratifying the complex milieu of cancer immunophenotypes into therapeutically-meaningful compartments that can serve as a guide for the study of cancer immuno-resistant mechanisms.

### From enhancing immunity to normalizing TIME

Historically, cancer immunotherapy has focused on amplifying tumor immunity above physiological levels, which is associated with clinical response in a minority of patients, in highly selected cancers (e.g., kidney and melanoma), and with off-tumor toxicities. It is becoming increasingly appreciated that many cancer patients have anti-cancer T cells, but the TIME can effectively suppress their immune response by harnessing immune homeostasis mechanisms to negatively regulate anti-cancer immunity or cell survival. As a result, cancer cells that can evade immune attack are naturally selected for survival. Hence, Lieping Chen and his colleagues have emphasized that, instead of enhancing the immune system, it is important to restore the function of the TIME [28]. The lessons we have learned from the failure of boosting immunity and the success of ICI development substantiate this notion of TIME normalization [29–31]. It is now crucial that we determine how to normalize the defects in TIME. In particular, targets for normalizing T1 (PD-L1^−^, TIL^−^) TIME remain to be discovered and validated. Searching for and defining such targets from T1 tumors are anticipated by Chen to be the next game changer in cancer immunotherapy. Chen reported one result that has already been gained from such efforts. He indicated that Siglec-15, mainly expressed by myeloid cells, may be a newly-defined T cell immune checkpoint target [32]. A first-in-class, first-in-human clinical trial of anti-Siglec-15 monoclonal antibody NC318 for the treatment of advanced cancers resistant to the current ICS has been ongoing since October 2018. Additional strategies aimed at normalizing the TIME by targeting myeloid cells and regulatory T cells are anticipated.

## Challenges and new perspectives in ICI development

### Simple addition: the traditional method of combination ICI development

To date, the standard approach taken by pharmaceutical companies to develop new ICI therapeutic regimens has been to combine two agents that each has shown single-agent activity. This approach has, in fact, yielded progress in some studies that have combined a chemotherapy agent with an ICI agent. As discussed by Roy Herbst from Yale University, the combination of chemotherapy with PD-1 inhibitor pembrolizumab as a first-line therapy for metastatic non-small cell lung cancer (NSCLC) was effective. Promising results were also yielded by the IMpassion130 study, which tested the combination of PD-L1 inhibitor atezolizumab and nano-particle albumin-bound chemotherapy agent nab-paclitaxel in metastatic triple negative breast cancer (TNBC) [33]. A similar conclusion can be drawn from the outcome of the Keynote-552 study, which tested the combination of pembrolizumab and chemotherapy in the neoadjuvant and adjuvant setting for resectable advanced triple negative breast cancer (TNBC) (ESMO 2019. Abstract LBA8_PR).

However, time will tell whether other chemotherapy/ICI pairing strategies will succeed or fail. Four anti-PD-1 antibodies that were developed by China-based biopharmaceuticals have been approved in China for two disease indications (Table 1). They have demonstrated similar anti-cancer activities as those developed in the USA although some of them demonstrated higher in vitro binding affinity to the PD-1. Three of them have been approved for treating classical Hodgkin’s lymphoma whereas one has been approved for treating melanoma [34–40]. None of them have been approved for more common types of malignancies such as NSCLC. By contrast, three anti-PD-1/PD-L1 antibodies that were developed by global pharmaceuticals have been approved to treat NSCLC. Although China’s anti-PD-1 antibodies lag behind in clinical developments as single agents, a strategic emphasis on developing anti-PD-1 antibody based combination immunotherapy has been made for these anti-PD-1 antibodies. In combination with chemotherapy agents, these IO agents are being tested as candidate first-line therapies for nasopharyngeal carcinoma, which is endemic in southern China. An investigator-initiated clinical trial testing the combination of chemotherapy and anti-PD-1 antibody camrelizumab in nasopharyngeal carcinoma showed an impressive response rate in 22 evaluable patients [41]. In this study, the combination of chemotherapy with camrelizumab yielded an overall response rate (ORR) of 91%, compared to the phase 1 ORR of 34% with camrelizumab monotherapy in chemotherapy-refractory nasopharyngeal carcinomas. These results provide a contrast to the findings of the Keynote-062 study, as discussed by Andrew Zhu from Massachusetts General Hospital and Lei Zheng from Johns Hopkins University. In Keynote-062, the combination of chemotherapy and pembrolizumab has failed to demonstrate clinical benefit in gastric and gastroesophageal carcinoma patients.

Another strategy for combination immunotherapy development is to combine an ICI and a tyrosine kinase inhibitor (TKI). Lawrence Fong from University of California at San Francisco and Lei Zheng from Johns Hopkins University discussed the results of the KEYNOTE-426 trial, which combined pembrolizumab and vascular endothelial growth factor receptor (VEGFR) inhibitor axitinib. This dual regimen enhanced progression-free survival (PFS), overall survival (OS), and overall response rate (ORR) over single-agent therapy with platelet-derived growth factor receptor (PDGFR)/VEGFR inhibitor sunitinib in treatment-naive advanced/metastatic renal cell carcinoma (mRCC) [42]. In another phase 3 study, PD-L1 inhibitor avelumab plus axitinib produced significantly prolonged PFS, OS, and ORR over sunitinib alone in treatment-naïve mRCC, irrespective of PD-L1 status [43]. Thus, the pembrolizumab/axitinib and avelumab/axitinib combinations have become the standard-of-care, first-line treatment option for mRCC. Another combination ICI/TKI therapy that has made it to first-line treatment status is the combination of pembrolizumab and multi-kinase inhibitor lenvatinib for advanced endometrial cancer [44].

Other ICI/TKI therapies have also shown potential. As reported at the 2019 Annual Meeting of the American Society of Clinical Oncology, the combination of anti-PD-1 monoclonal antibody nivolumab and angiopoietin receptor/VEGFR inhibitor regorafenib was found to yield promising objective responses in chemotherapy-refractory gastric adenocarcinoma and colorectal adenocarcinoma. Similarly, camrelizumab combined with VEGFR2 inhibitor apatinib in advanced HCC, gastric junction cancer, or esophagogastric junction cancer produced a 50% ORR, a 93.8% disease control rate (DCR), and a PFS period of 7.2 months in HCC [45]. All of these metrics were better than those produced by camrelizumab alone. Furthermore, these results are comparable to, if not better than, those of a clinical trial testing the combination of pembrolizumab and VEGFR inhibitor lenvatinib in HCC, as well as those of a clinical trial testing the combination of nivolumab and regorafenib in gastric and gastroesophageal carcinoma. The combination of anti-PD-1 antibody toripalimab and axitinib in treating mucosal melanoma has also shown encouraging results [46]. Taken together, these studies indicate that combination ICI/TKI therapy may be an effective strategy, even though its mechanism of action remains unknown. It is possible that one of the two treatments stablizes disease, allowing more patients to respond to the other treatment. Nevertheless, combination ICI/TKI therapy has indeed been built on strong evidence of the clinical benefit of ICI and TKI, either as single-agents or in combination. Whether this strategy is able to overcome the challenge of ICI-resistant tumors remains to be investigated.

A third combination therapy strategy is to combine agents targeting different immune checkpoint pathways. The combination of anti-CTLA-4 and anti-PD-1/PD-L1 antibodies has shown augmented efficacy versus monotherapy in several cancers, but mainly in those that are known to have responded to anti-PD-1/PD-L1 antibodies as a single agent. As discussed by Andrew Zhu, this approach is standard-of-care for melanoma [47] and renal cell carcinoma [48], and it is considered to be a promising treatment option for hepatocellular carcinoma. More recently, the combination of nivolumab and CTLA-4 inhibitor ipilimumab was shown to significantly enhance OS when compared to first-line chemotherapy in metastatic NSCLC, regardless of PD-L1 status [49]. Whether combination nivolumab/ipilimumab is effective in cancer types that are primarily resistant to anti-PD-1/PD-L1 ICIs remain questionable.

### Lessons learned from the development and application of immune checkpoint inhibitors

ICIs continue to receive approval for new indications; however, these indications are underpinned by clinical proof-of-concept data that were produced before 2016. Besides CAR-T therapies, no new immunotherapy strategies have been brought into standard clinical practice after the initial emergence of ICIs. While numerous clinical trials on new immunotherapeutic agents have been conducted, including many that combine new agents with existing ICIs, the majority of these clinical trials have not shown promising results, and some have shown disappointing results. For example, the field learned a lesson from the results of the Phase-III ECHO-301 trial, which tested the combination of epacadostat with pembrolizumab in metastatic melanoma [50]. Epacadostat is an orally-bioavailable inhibitor of indoleamine 2,3-dioxygenase 1 (IDO1), a rate-limiting enzyme in tryptophan catabolism that plays important roles in immune suppression [51]. Despite encouraging early-phase data [52, 53], the ECHO-301 trial did not demonstrate that the combination of epacadostat and pembrolizumab yielded a superior outcome to pembrolizumab alone [50]. The failure of the ECHO-301 trial calls for a better rationale when designing trials for combination immunotherapy. Several strategies for improvement were discussed at the 2019 China Cancer Immunotherapy Workshop.

Roy Herbst presented the long-term follow-up results from both the CA209-003 and Keynote-001 studies, which have clearly documented the long-term survival benefit of nivolumab and pembrolizumab, respectively, in NSCLC [54, 55]. Herbst reviewed the studies that have demonstrated the correlation between higher frequencies of tumor-infiltrating lymphocytes (TIL) and better survival following ICI treatment. However, more specific biomarkers to define TIL subtypes are needed to better predict tumor sensitivity to ICIs. Concerning combination immunotherapy for NSCLC, Herbst highlighted the success of combining chemotherapy and pembrolizumab as a first-line therapy for NSCLC that is not a candidate for targeted therapies [56]. He suggested that future strategies to enhance the efficacy of combination therapy should include the following: (1) reducing tumor bulk to improve the T cell:tumor target ratio, (2) reducing T cell inhibitory substances produced by the tumor, (3) altering tumor barriers (i.e., vasculature/pressure) to T cell penetration, (4) using cancer vaccines to sensitize T cells and antigen-presenting cells (APCs), and (5) altering T cell signaling/gene expression to enhance the production of T cell attractants. In addition, Herbst noted that biomarker-driven studies such as the database-rich Lung-MAP study, the Keynote-495/KeyImPaCT NSCLC “umbrella” study of multiple pembrolizumab-based combinations, and a durvalumab (PD-L1 inhibitor)-based study of locally advanced NSCLC with biomarker correlation, could open the door for individualized combination immunotherapy for NSCLC patients.

In contrast, Andrew Zhu pointed out that ICIs are only indicated for a small percentage of gastrointestinal cancer patients. The Checkmate-040 study led to the approval of nivolumab [57], and the Keynote-224 study led to the approval of pembrolizumab [58], as second-line therapies for HCC. Both antibodies produced a durable response in the majority of treatment-responsive patients. However, the Phase-III CheckMate-459 study comparing nivolumab to multi-kinase inhibitor sorafenib as a first-line therapy for HCC failed to meet the primary endpoint. Nevertheless, the combinations of durvalumab with CTLA-4 inhibitor tremelimumab, ipilimumab with nivolumab, and atezolizumab with VEGF-A inhibitor bevacizumab have all demonstrated promising ORRs. The Phase-Ib study of pembrolizumab and lenvatinib as a first-line therapy for HCC showed an impressive 42.2% ORR. All considered, these studies suggest that combination immunotherapy may become a major platform for treating gastrointestinal cancer patients. After the initial approval of nivolumab and pembrolizumab as third-line therapies for gastric or gastroesophageal cancers, however, single-agent ICI therapies have encountered multiple failures in attempting to expand their indications to first-line and second-line settings. Nevertheless, both pembrolizumab and nivolumab can prolong OS when compared to second-line chemotherapy in patients with squamous cell carcinoma of the esophagus [59], which would be anticipated in this relatively high mutational-burden tumor. Earlier, ICIs had been approved for microsatellite instability (MSI)-high colorectal cancers, which constitute approximately 5% of metastatic colorectal cancers, as well as other MSI-high gastrointestinal malignancies which constitute less than 1‑2% of their respective types of tumors [60, 61]. Therefore, the majority of gastrointestinal cancers still do not respond to ICIs as a single agent, making combination immunotherapy strategies highly desirable.

From an immunology perspective, Lei Zheng shared his viewpoints on the lessons learned from failed IO clinical trials. He summarized five major reasons for the failures, based on the tendency of the field to make unsubstantiated assumptions. The first reason is the assumption that two ICIs are better than one. A lesson in this reason for failure came from clinical trials that combined an anti-PD-1 antibody with an IDO inhibitor or other ICIs (such as anti-LAG3 antibody) as discussed above. The second reason is the assumption that there is synergy between ICIs and chemotherapy. This was exemplified in the outcome of the Keynote-062 study, where pembrolizumab in combination with chemotherapy failed to demonstrate a clinical advantage in gastric and gastroesophageal cancers. The third reason is the assumption that if a combination works for one cancer type, it would also work for other cancer types. The lack of treatment efficacy in Keynote-062 is disparate from the success of using combination chemotherapy and pembrolizumab in NSCLC. The fourth reason is premature conclusions based on results from studies with small sample sizes. This is best exemplified by a Phase-Ib study of nivolumab in combination with NKTR-214, an IL-2 receptor (IL-2R) agonist with biased activation through the IL-2Rβ/γ subunits [62]. This early study had shown a high ORR, which decreased to a lower ORR with more patients enrolled. A study with a small sample size must be interpreted with caution. The only way to prove the effectiveness of a combination therapy is to conduct a well-controlled, randomized study with a sufficiently large sample size to draw the definitive statistical conclusion. The fifth reason is underestimation of the number of immune “defects” that would need to be normalized. Although inflamed tumors may only have a single “defect”, such as T cell exhaustion due to activation of immune checkpoints, non-inflamed tumors often have multiple “defects” in the cancer-immunity cycle [63].

## Fast and furious development of cell therapy

Although a paradigm shift from immunity enhancement to normalization of TIME may be advisable in IO development for solid tumors, immunity enhancement remains a mainstay therapeutic strategy for hematologic malignancies. CD19-targeted CAR-T cells for B cell neoplasms have opened up a new era in synthetic cancer immunotherapy [64]. There are two approved CD19-CAR-T cell platforms: tisagenlecleucel (Kymriah) and axicabtagene ciloleucel (Yescarta). Besides CD3ζ chain, Tisagenlecleucel uses CD137 (4-1BB) as additional co-stimulating signal (COS), while axicabtagene ciloleucel uses CD28 a COS. Both agents utilize a single chain anti-CD19 fragment to target malignant B cells. Tisagenlecleucel is approved for the treatment of patients up to 25 years of age with B cell precursor acute lymphoblastic leukemia (ALL) that is refractory to standard therapy or in at least second relapse. Both agents are indicated for the treatment of relapsed or refractory large B cell lymphoma.

### Cutting-edge developments in adoptive cell therapy in the USA

Cassian Yee from the University of Texas MD Anderson Cancer Center reviewed more than 20 types of antigen-specific T cells from the peripheral blood of cancer patients for adoptive transfer therapy [65]. Yee presented the experience of his research group to use endogenous T cell (ETC) therapy alone to put advanced melanoma into long term remission. Yee’s group was also the first to combine ICI therapy with human central memory T cell ETC therapy to treat advanced solid tumors, which yielded astonishingly good responses in some patients [66]. In addition, Yee reported on several investigator-initiated clinical trials in multiple solid tumor types.

Elizabeth Budde from the City of Hope National Medical Center presented long-term follow-up data from several major CAR-T trials in lymphoma, showing approximately 40% PFS at 2 years after CAR-T treatment [67]. Budde also provided an update on the real-world data concerning the two FDA-approved CD19 CAR-T products, which reflects a large fraction of patients who would have been ineligible for the original CAR-T clinical trials. It is reassuring that the real-world response rate, the severity of cytokine release syndrome (CRS), and neurotoxicity were comparable with the results from the clinical trials, indicating that CAR-T can be successfully used to provide clinical benefit in more patients at CAR-T certified medical centers [68]. Multiple myeloma is no doubt one of the most active areas of CAR-T development. CAR-T therapy targets for multiple myeloma include BCMA, CD19, SLAMF7 (CS1), NKG2D, CD56, CD70, CD38, CD138, CD44v6, and IgκλC. Among them, BCMA is the most studied [69, 70]. Two BCMA-targeted CAR-T clinical trials (bb2121 and LCAR-B38M) evidenced a 50‑74% CR rate, a 10.8‑16-month duration of response, and an 11.8‑15-month PFS [71, 72]. On the other hand, targets for CAR-T therapy in AML include CD123, CD33, NKG2D, Lewis Y, FLT-3, CLL-1, CD44v6, IL1-RAP, and TIM-3 [73, 74]. CD123 is the most-studied target. Budde is conducting CAR-T cell therapy targeting CD123 in AML patients at the City of Hope, and promising outcomes have already been observed in some patients [75, 76].

Yangbin Zhao from the University of Pennsylvania discussed his group’s efforts with genetic engineering to improve T cell therapy for solid tumors. Zhao pointed out that the ideal T cells for cellular therapy might be effector memory T (T_EM_)-like cells with excellent proliferation and long-term in vivo persistance [77, 78]. Zhao’s update focused on the ongoing first US trial with universal CAR-T cells that were genetically edited using CRISPR/Cas9 technology to eliminate their expression of endogenous TCRs and PD-1. This universal CAR-T cell product targets ESO-1 in solid tumors. Innovative designs of CAR-T cells to enhance their efficacy and function were described, including CAR-T cells with co-expression of a fusion protein between extracellular domain of PD-1 and transmembrane/signaling domain of CD28 to rescue them from hypofunction and enhance their tumor-killing effects [79]. Zhao also provided a briefing on a new strategy to ectopically express a dominant-negative transforming growth factor-beta receptor (TGFβR), thereby removing a negative control mechanism of CAR-T function [80].

Finally, Hongtao Liu from the University of Chicago discussed the management of CAR-T toxicities. Liu discussed the newly-published American Society for Transplantation and Cellular Therapy (ASTCT) consensus grading system for CRS and neurologic toxicity associated with immune effector cells. The new grading relies heavily on clinical parameters and presentation, which could be easily used at the bedside [81]. In addition, Liu discussed the risk factors for CRS and neurologic toxicities such as immune effector cell-associated neurotoxicity syndrome (ICANS), as well as recent studies that might change the management of these toxicities. Liu presented new findings from two separate groups on the critical roles of IL-1 receptor (IL-1R) and granulocyte-macrophage colony-stimulating factor (GM-CSF) in CRS and ICANS, providing a rationale to block the IL-1R pathway using the IL-1R antagonist, Anakinra [82, 83], and to neutralize GM-CSF, in order to control and prevent CRS and ICANS during CAR-T treatment [84].

### China update: rapid catch-up in the space of cell therapy

Investigators in China have also made marked progress in immunotherapy, particularly in the realm of adoptive cell transfer therapy. Jun Ren from Beijing Shijitan Hospital of Capital Medical University discussed autologous dendritic cell and cytokine-induced killer cell (DC-CIK) cellular immunotherapy, as well as combination DC-CIK and chemotherapy [85, 86]. Correlation of immunological biomarkers with clinical efficacy and clonal diversity of the TCRβ repertoires was reported [87]. The combination of tumor vaccines and hyperthermia therapy with cellular therapy also has generated promising clinical responses [88]. Several clinical trials that combine anti-PD-1 antibody with cellular immunotherapy have been initiated for several tumor types, including metastatic NSCLC, SCLC, HCC, and other advanced solid tumors.

Weidong Han from Chinese PLA General Hospital presented his group’s clinical results from therapy using CD19^+^CD22^+^ dual-targeted CAR-T cells (CD19^+^CD22^+^ CAR-T), which suggests a general rationale for building dual-targeted CAR-T cells. One ALL patient who failed CD19^+^CAR-T therapy was able to enter long term CR with CD19^+^CD22^+^CAR-T therapy [89]. CD19^+^CD22^+^CAR-T therapy has strong potency with limited CRS and neurotoxicity. In 46 evaluable refractory/resistant NHL patients, the ORR (CR + PR) reached 97.8% and the CR reached 82.3% after 1 month post-CAR-T infusion; at 6 months post-CAR-T infusion, the OOR was 69.2%, which was favorable in comparison with the results from large historical studies using single-target CD19^+^ CAR-T. The main reasons for disease relapse/progression included the following: (1) loss of the target antigen on the leukemia cells, (2) low level or loss of CAR-T cells after infusion, (3) CAR-T suppression by the negative regulators in the TIME, and (4) acquired resistance to weak CAR-T cell efficacy. Han also noted that some initial PR patients could exhibit delayed CR with longer follow-up.

Jun Zhu from Peking University Cancer Hospital provided an update on his group’s CAR-T product (MC-19PD1 CAR-T), which contains a PD-1/CD28 common gene chimerism for switching the suppressive signal of PD-1 to an activating signal that enhances CAR-T efficacy. Their pilot trial enrolled 17 relapsed/refractory lymphoma patients, including 15 patients with PD-L1^+^ lymphoma, which typically had poor or no response to the CAR-T products from Kite and Novartis. The ORR was 58.8% (CR rate of 41.2%), which were associated only with grade 2 CRS and grade 1 neurotoxicity. Zhu also discussed approaches to modify the hinge and transmembrane regions of CD8α in a CAR-T construct, in order to decrease the risk of CRS without compromising the anti-tumor efficacy of the modified CAR-T cell [90].

## Neoantigen-based vaccination: a promising strategy to boost immunotherapy

An effective cancer-immunity cycle needs to be initiated by an innate immune response to drive the antigen presentation and priming process. Thus, an IO strategy that has emerged is to stimulate a strong innate immune response that will support robust antigen presentation and innate immunity. Neoantigen-based vaccination is one approach to this strategy, and it was discussed by several investigators at the Workshop.

### In situ vaccination can potentiate the clinical response to ICI therapy

Tony Ribas from University of California at Los Angeles elaborated on the mechanisms of acquired resistance to ICIs: (1) defects in antigen presentation, and (2) defects in the IFN-γ signaling pathway [91, 92]. In situ vaccination has emerged as a candidate method for overcoming these defects, by facilitating TIME priming. Specifically, toll-like receptor 9 (TLR9) agonists [93], oncolytic virus [94], and IL-2 receptor agonists [95], represent three major targets for in situ vaccination. Clinical trials using these approaches to prime the melanoma TIME and enhance the sensitivity of anti-PD-1 antibodies have been conducted. Preliminary results from these studies support using this novel strategy in overcoming the resistance to ICIs through activating T cells directly by activating type-I cytokine receptors or indirectly through activating innate immune responses.

Ronald Levy from Stanford University shared with the audience his recent research on the in situ vaccination strategy. Levy discussed intratumoral injection of the CpG oligodeoxynucleotide (CpG) as an in situ therapeutic vaccination to boost anti-cancer immunity. Unmethylated CpG commonly exists in microbial genomes, but rarely in vertebrates [96]. Therefore, CpG is recognized via TLR-9 expressed by APCs, including dendritic cells and B cells [97], which activates both innate and adaptive immune responses. In tumor-bearing mice, CpG induced an anti-tumor response only after direct intratumoral injection. When intratumoral vaccination with CpG was combined with low dose radiation during a multi-center Phase-I/II clinical trial in patients with low-grade B-cell lymphoma, durable responses were observed at distant tumor sites [98]. With this success, several other combinations were tested both in the preclinical murine setting as well as in clinical trials. For example, Ibrutinib, a BTK and ITK inhibitor that can suppress myeloid-derived suppressor cells and regulatory T (T_reg_) cells, produced synergistic anti-tumor activity when combined with CpG and low-dose radiation. In some cases, complete remission was observed in the treated lesions as well as distant lesions that were not treated with CpG. Flow cytometry and single-cell sequencing with paired biopsy specimens obtained pre- and post-treatment showed a decrease in tumor B cells post-treatment, while normal NK, B, and T cells increased. In addition, CpG vaccination not only stimulated an immune response but also induced the expression of OX40, also known as tumor necrosis factor receptor superfamily member 4, and a secondary co-stimulatory molecule expressed on T_regs_ and activated T cells. The combination of CpG vaccination and anti-OX40 therapy enhanced the anti-tumor immune response and eliminated established lymphoma as well as solid tumors in mice. In fact, this combination was more effective than the combination of CpG and anti-PD-L1 antibody [99]. With these promising results in hand, two clinical trials are currently ongoing: (1) combination therapy with CpG, OX40 agonist, and low-dose radiation for non-Hodgkin’s lymphoma and (2) combination therapy with CpG and OX40 agonist in all cancer types.

Liang Deng from Memorial Sloan Kettering Cancer Center discussed a novel virotherapy based on vaccinia virus, another approach to the neoantigen-based in situ vaccination strategy. Similar to CpG, oncolytic virus is another kind of in situ therapy that can stimulate cancer immunotherapy as outlined in Fig. 1 (outermost circle). Oncolytic virus triggers an antitumor immune response through induction of immunogenic cell death, release of tumor-associated antigens (including damage-associated molecular patterns (DAMPs)), alteration of an immunosuppressive TIME, and promotion of dendritic cell maturation and antigen presentation. Hence, localized oncolytic virus can convert non-immunogenic “cold” tumors into immunogenic “hot” ones, inducing tumor infiltration by immune cells and overcoming resistance to ICIs [100]. T-VEC is a replication-competent Herpes Simplex 1 (HSV-1) virus that expresses human GM-CSF (hGM-CSF). It was approved in the USA for treatment of advanced melanoma in 2015, making it the first oncolytic virus approved for this indication. However, compared to hGM-CSF control therapy, intratumoral injection of T-VEC only improved OS by 4.4 months [101]. To further improve clinical efficacy, T-VEC is being tested in combination with immune checkpoint inhibitors, such as anti-CTLA-4 antibody [102]. On the other hand, modified, attenuated vaccinia virus Ankara (MVA) is a new generation of smallpox vaccine that serves as a promising vaccine vector for infectious diseases and cancer. It has a deletion of 30 Kb from the parental vaccinia genome, which inhibits replication of the virus in mammals [103]. Intratumoral injection of heat-inactivated MVA induces innate immunity via the STING pathway, which enhances tumor antigen presentation, promotes dendritic cell maturation, stimulates naïve T cell priming, increases tumor-specific T cell expansion and migration, and boosts cytotoxic T lymphocyte (CTL)-mediated killing of cancer cells [104–106]. The anti-tumor effect of heat-inactivated MVA requires CD8^+^ T cells, and the long-term anti-tumor memory response requires CD4^+^ T cells. To generate the next generation of MVA with still greater efficacy, MVA with deletion of C7L (MVAΔC7L) was generated. MVAΔC7L can induce much higher levels of type I interferon, proinflammatory cytokines, and chemokines [105, 107]. Additionally, MVA can be further engineered to express Flt3L, which is a growth factor for CD103^+^ and plasmacytoid dendritic cells, and OX40L, which serves as co-stimulatory ligand for OX40 expressed by T cells. After intratumoral injection, Flt3L- and OX40L-expressing MVAΔC7L induced more CD8^+^ and CD4^+^ T cells responding in distant, non-injected tumors, and synergized with anti-PD-L1 antibody in multiple mouse tumor models as compared to heat-inactivated parental MVA. Currently, the Memorial Sloan Kettering Cancer Center has two vaccinia-based vectors: (1) recombinant MVA that expresses a non-replicative, safe immune activator, activates multiple innate immune pathways (including cGAS/STING), and can be used for intratumoral injection as a monotherapy or in combination with ICIs; (2) an oncolytic vaccinia platform that is replication-competent, has the capability to express large proteins (e.g., antibodies against immune checkpoint molecules), enhances anti-tumor activity and reduces immune-related toxicities.

### Personalized neoantigen-based vaccination as a novel combination immunotherapy in cancer

David Reardon from Dana-Farber Cancer Institute and Harvard Medical School discussed peptide-based neoantigen vaccination as a personalized cancer immunotherapy. All cancer cells harbor genetic alterations, including missense, deletion, frame shift, and gene fusion mutations, which can generate tumor neoepitopes. If these neoepitopes can be presented to MHC molecules for T cell recognition, in theory, they can be formulated to make neoantigen-specific cancer vaccines [18]. Previous studies have shown that high neoantigen load is associated with better immunotherapy outcomes. Most investigators use three parallel steps to generate a personalized neoantigen vaccine: (1) DNA from normal cells is sequenced to determine the human leukocyte antigen (HLA) type; (2) paired normal and tumor DNAs are sequenced to detect somatic mutations; and (3) tumor mRNA is sequenced to determine gene expression. Information from these three approaches is combined to predict personal HLA-binding peptides which will guide the manufacturing of candidate neoantigens for personalized vaccine. In the first study with six patients with melanoma as reported by Reardon, four had no cancer recurrence at 25 months after vaccination while the remaining two patients had recurrence, but achieved complete tumor regression after subsequent anti-PD-1 therapy [108]. Results using the enzyme-linked immune absorbent spot (ELISPOT) assay showed significant ex vivo IFN-γ responses against a neoepitope pool. Neoantigen vaccination elicited polyfunctional de novo CD4^+^ and CD8^+^ T cell responses against neoepitope pools and induced an anti-tumor T cell response that discriminated between mutated antigens and the corresponding wild type epitopes. Having obtained these exciting results with neoantigen vaccination in melanoma, a glioblastoma NeoVax trial was designed that included approximately 20 synthetic long (i.e., 20-30-mer) peptides per patient [109]. This was an open-label, Phase-I study that included newly-diagnosed glioblastoma patients with MGMT-unmethylated tumors. The vaccination schedule included five injections of high antigen exposure during the first 4 weeks, followed by two boost vaccinations at weeks 12 and 20. The therapy was very well tolerated with no dose-limiting toxicity, and there were no dose delays. Multiple de novo, polyfunctional T cell responses were noted primarily against mutant and not wild-type peptides among patients not treated with dexamethasone. When relapsed tumor tissue was analyzed to assess post-vaccination changes, increased CD4^+^ and CD8^+^ T cell infiltration was observed in dexamethasone-untreated patients, but not in dexamethasone-treated patients. Infiltrating T cells detected at the intracranial tumor site were neoantigen-specific. With a median follow-up of 31.5 months, the median PFS was 14.2 months and the median OS was 29.0 months. It can therefore be concluded that the generation of personalized vaccines based on individual patient mutation profiles and HLA types is feasible, and that these vaccines are well-tolerated and can induce neoepitope-specific immune responses. A new clinical trial has been planned, which will combine NeoVax with pembrolizumab for patients with newly-diagnosed GBM.

Finally, Robert Schreiber from Washington University discussed approaches in characterizing CD8^+^ and CD4^+^ T cell epitopes and TCR repertoire in murine tumor models [110]. Identification and characterization of neoantigen-specific T cells within tumors is anticipated to lead to a new wave of adoptive T cell therapies, which can be actively developed as a personalized immunotherapy strategy in tandem with neoantigen-based vaccine therapy.

## Metabolic reprogramming: the key for sustained T cell effector function in cancer immunotherapy

Although the innate immune response and antigen presentation are crucial for the initiation of an anti-tumor immune response, effective antitumor immunity is often not sustained due to the dysfunctional status of effector T cells due to both T cell intrinsic and extrinsic mechanisms. In the 2019 China Cancer Immunotherapy Workshop, several cutting-edge unpublished preclinical and clinical studies were reported, which demonstrated the role of metabolism in anti-tumor immune modulation.

### Reprogramming of amino acid metabolism to sensitize tumors for immunotherapy

Weiping Zou from University of Michigan elucidated how ferroptosis, an iron-dependent, peroxidation-induced type of cell death, regulates the immune system [111, 112] and functions as an important effector T cell-mediated pathway of cancer cell death. Zou’s research has found that IFN-γ sensitizes and promotes tumor cell ferroptosis, whereas ferroptosis inhibition attenuates anti-tumor immunity. Zou’s group further dissected the mechanisms of IFN-γ-regulated tumor cell ferroptosis and found that IFN-γ regulates tumor ferroptosis by targeting SLC7A11 and SLC3A2. Both SLC7A11 and SLC3A2 are cystine transporters that are responsible for cystine uptake, glutamate release, and glutathione maintenance. Thus, the reprogramming of amino acid metabolism is crucial for immune-mediated tumor cell death, and targeting ferroptosis may sensitize tumors to immune attack.

### Targeting the adenosine pathway to potentiate cancer immunotherapy

Extracellular adenosine has significant immunosuppressive effects on both effector immune cells and immunosuppressive regulatory cells [113, 114]. Adenosine is metabolized through dephosphorylation of ATP by CD39 and CD73, which are highly expressed on stromal and immune cells in the TIME [115]. Interaction of adenosine with its receptor blocks T cell activation and promotes myeloid suppression. Hence, targeting adenosine and other molecules upstream and downstream of its pathway may restore anti-cancer immunity (Fig. 1, outermost circle). Lawrence Fong from University of California San Francisco discussed strategies to potentiate cancer immunotherapy through targeting the adenosine pathway. CPI-444 is an oral small molecule antagonist of the adenosine 2A receptor (A2AR) [116]. A Phase-I/Ib clinical trial with oral CPI-444 alone or CPI-444 in combination with atezolizumab is currently ongoing in patients with renal cell carcinoma, non-small cell lung cancer, melanoma, triple-negative breast cancer, or other cancer types (unpublished data). These patients have exhibited progressive disease on prior therapy, including immunotherapy, and they were not selected based on PD-L1 expression. So far, this regimen has been tolerated very well. A2A inhibition alone or in combination with atezolizumab has led to clinical responses in both atezolizumab-naïve as well as atezolizumab-resistant/refractory patients. CPI-444 treatment induces CD8^+^ T cell infiltration into tumor sites, and the adenosine gene signature is associated with tumor response to therapy. Since adenosine is converted from AMP by CD73, another clinical trial is currently ongoing with the humanized anti-CD73 antibody CPI-006, which blocks CD73 catalytic activity and has agonistic immunomodulatory activity on CD73^+^ cells. CPI-006 has been well-tolerated at the dose of 12 mg/kg, can completely inhibit CD73 enzymatic activity in tumor biopsies, and can induce serum pro-inflammatory cytokines. In conclusion, targeting the adenosine pathway can potentially reverse an immunosuppressive TIME and potentiate the anti-tumor response to ICIs.

### Discovering and targeting the RNA metabolic switch between regulatory and effector T cells

RNA-binding proteins are important regulators of RNA biology (splicing, stability, etc.) and protein translation. Recently, it was found that these proteins play critical roles in the regulation of gene expression upon T cell activation. One member of the RNA-binding proteins, the poly-C-binding protein 1 (PCBP1), binds to DNA and RNA and controls protein expression. In addition, its phosphorylation status can determine its RNA binding function [117]. In a chronic immunosuppressive setting such as cancer, the phosphorylation of PCBP1 induced by TGF-β can affect expression of proteins such as moesin [118], and it can regulate T cell differentiation and the immune response. Zihai Li from The Ohio State University reported his group’s unpublished data on the roles of PCBP1 in stabilizing effector T cell (T_eff_) function and suppressing the T_eff_-T_reg_ commitment program (Ephraim Ansa-Addo and Zihai Li, unpublished). However, once T_regs_ have differentiated, PCBP1 knockdown can no longer reverse this program back to the T_eff_ program. Consequently, genetic disruption of PCBP1 in T cells exacerbates tumor growth. However, melanoma with low PCBP1 expression responds better to anti-PD-1 therapy, suggesting that, in this ICI-sensitive tumor, the T_eff_-T_reg_ commitment program sensitizes tumors for ICI treatment. Li also showed that high PCBP1 expression is associated with low expression of immune checkpoint signals in melanoma. Therefore, it will be interesting to see the expression levels of PCBP1 in ICI-resistant, cold tumors, and examine how this plays a role in the T_eff_-T_reg_ commitment program and ICI resistance.

## Conclusions and future perspectives

The development of cancer immunotherapy for ICI-resistant cancers has been a challenge. Current ICI-based combination therapy strategies have achieved some, albeit limited, success. A deep understanding of TIME biology in the IO field is necessary for generating next generation immuno-oncology therapeutic strategies. ICIs, CAR-T therapy, adoptive cell therapy, and other anti-tumor immunity enhancement approaches, will continue to lead the way in the clinical IO space. However, new classes of immunotherapy are emerging. These new classes aim at normalizing the “defective” TIME by targeting immunosuppressive components unique to the tumors, priming effector T cells by in situ oncolytic therapy, broadening effective T cell repertoire with multi-valent neoantigen-based vaccines, modulating metabolic programming for sustained T cell function and promoting effective immune-mediated cell death. All together, these emerging strategies point towards a promising new wave of cancer immunotherapies that may allow us to surmount the limitations of previous ones.

## Data Availability

The authors agree to open access for this publication.

## Abbreviations

BCMA: B cell maturation antigen
CAHON: Chinese American Hematologist and Oncologist Network
CAR-T: T cells expressing chimeric antigen receptor
CSCC: Cutaneous squamous cell carcinoma
CTLA-4: Cytotoxic T Lymphocyte-Associated Protein 4
dMMR: DNA mismatch repair deficiency
ETC: Endogenous T cells
HLA: Human leukocyte antigen
ICI: Immune checkpoint inhibitor
IDO: Indoleamine 2,3-dioxygenase
NMPA: China National Medical Product Administration
MSI-H: Microsatellite instability high
NSCLC: Non-small cell lung cancer
PD-1: Programmed cell death protein 1
PD-L1: Ligand 1 from programmed cell death protein 1
SITC: Society of Immunotherapy of Cancer
US FDA: The United States Food and Drug Administration
TIME: Tumor immunity in the microenvironment
